# Proteomic Insights into Human Limbal Epithelial Progenitor-Derived Small Extracellular Vesicles

**DOI:** 10.1007/s12015-025-10877-w

**Published:** 2025-04-16

**Authors:** Moritz Vincent Braunsperger, Gottfried Martin, Tabea Herzig, Isabell Kußberger, Andreas Gießl, Stefan Steimle, Ursula Schlötzer-Schrehardt, Günther Schlunck, Thomas Reinhard, Naresh Polisetti

**Affiliations:** 1 Eye Center, Medical Center - Faculty of Medicine, University of Freiburg, Killianstrasse 5, 79106 Freiburg, Germany; 2 Department of Ophthalmology, University Hospital Erlangen, Friedrich-Alexander-University of Erlan-gen-Nürnberg, Schwabachanlage 6, D-91054 Erlangen, Germany; 3 Cryo-EM Facility (CEF), University of Freiburg, Albertstrasse 21, 79104 Freiburg, Germany; 4 Institute of Physical Chemistry, University of Freiburg, Albertstrasse 21, 79104 Freiburg, Germany

**Keywords:** Limbal epithelial progenitor cells, Small extracellular vesicles, Exosomes, Limbal melancoytes, Limbal mesenchymal stromal cells, Limbal stem cell niche, Proteomics

## Abstract

Limbal epithelial stem/progenitor cells (LEPC), supported by limbal mesenchymal stromal cells (LMSC) and limbal melanocytes (LM) within a specialized niche, are responsible for maintaining the corneal epithelium. Small extracellular vesicles (sEV) emerged as critical mediators of intercellular communication in various stem cell niches, yet their role in maintaining human limbal niche homeostasis remains poorly understood. In this study, tangential flow filtration and size exclusion chromatography were used to isolate sEV from LEPC-, LMSC- and LM-conditioned media. The isolated sEV from LEPC exhibited properties characteristic for sEV as confirmed by nanoparticle tracking analysis for size and concentration, by electron microscopy for morphology, and by western blot analysis of canonical EV markers including the cell-specific protein (cytokeratin 17/19). Quantitative and comparative proteomic profiling revealed distinct molecular signatures of LEPC-derived sEV, enriched in factors associated with keratinocyte development, extracellular matrix organization, and niche regulation. These findings suggest that LEPC-derived sEV may serve as important signaling mediators within the limbal niche microenvironment, though additional studies are needed to determine their specific functional roles in maintaining niche homeostasis.

**Graphical Abstract:**

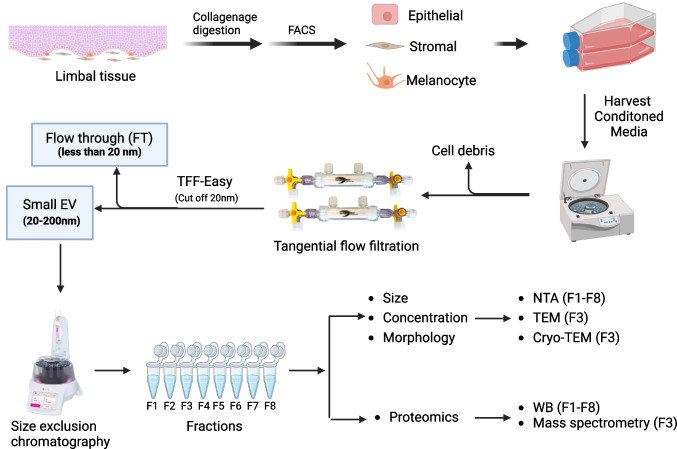

**Supplementary Information:**

The online version contains supplementary material available at 10.1007/s12015-025-10877-w.

## Introduction

The cornea is a specialized tissue, notable for its transparency and avascularity, which are essential for allowing light to pass through and enabling clear vision. Its outermost layer, the corneal epithelium, depends on constant homeostasis and regeneration to maintain corneal transparency and visual acuity. These processes are sustained by the activity of limbal epithelial progenitor cells (LEPC), located within the basal epithelial layers of the corneoscleral limbus [1]. The functionality and self-renewal capacity of LEPC are tightly regulated by a specialized niche microenvironment characterized by distinct physical parameters, molecular signals, and the presence of several supportive cell types, including limbal mesenchymal stromal cells (LMSC) and limbal melanocytes (LM), as well as a distinct extracellular matrix(ECM) composition [2–6]. Crosstalk between LEPC and their niche components occur through direct cell-cell interactions and paracrine mechanisms, including those mediated by soluble factors and extracellular vesicles (EV), which are vital for the stem cell maintenance and activation [5, 5–10].

Emerging evidence suggests that EV play a critical role in intercellular communication by transporting proteins, lipids and nucleic acids to recipient cells via paracrine and autocrine signaling [11–13]. This cargo delivery can induce phenotypic changes and reprogram recipient cells, highlighting the functional versatility of EV in niche dynamics [11–14]. EV are lipid bilayer-bound vesicles secreted by all cell types into extracellular space, characterized by their heterogeneity in size, composition, and function [15, 16]. Generally, EV can be categorized based on size, transmembrane marker expression, and biogenesis pathways into small EV (sEV)/exosomes (40–150 nm), microvesicles (100–1000 nm), and apoptotic bodies (1000–5000 nm). Among these, sEV, derived from endosomes, have garnered significant attention as functional mediators of intercellular communication, influencing both physiological and pathological processes [17, 18]. Moreover, sEV are of considerable clinical interest due to their potential applications in disease diagnosis via liquid biopsy and therapeutic interventions [19]. In corneal biology, the research on EV is in its early stages. Notably, LEPC-derived EV have a pronounced effect on stromal cell wound healing, proliferation, and stem cell marker expression [20]. EVs derived from immortalized corneal epithelial cells have shown a protein cargo rich in ECM proteins and induced myofibroblast differentiation of stromal cells [21, 22]. Moreover, murine corneal epithelial cell-derived exosomes induced vascular endothelial cell proliferation and ex vivo ring sprouting [22]. These findings suggest a potential role for limbal/corneal epithelial cell-derived exosomes in corneal wound healing and neovascularization. Recently, Yeung and colleagues [23] demonstrated different molecular compositions of EV derived from corneal epithelium and stroma, though their analysis was limited to corneal cell types. However, comprehensive characterization of primary LEPC-derived sEV remains sparse, and comparative proteomics data across major limbal cell types (LEPC, LMSC, and LM) are lacking.

To address these gaps, sEV were isolated from conditioned media (CM) of LEPC, LMSC and LM using tangential flow filtration (TFF) and size exclusion chromatography (SEC). The isolated sEV populations were characterized by nanoparticle tracking analysis (NTA), Western blot (WB), and electron microscopy. Notably, isolated LM-sEV and LMSC-sEV were included only in selected experiments, while other analyses utilized previously published data from our earlier work, as detailed in the respective experimental sections. Additionally, the protein cargo of LEPC-sEV was determined by mass spectrometry, and the proteomics data were compared to data on LMSC and LM-sEV protein cargo we reported earlier [24].

## Materials and Methods

### Tissue Samples

Human donor corneoscleral tissue with appropriate research consent was provided by the Lions Cornea Bank Baden-Württemberg after retrieval of transplants for Descemet membrane endothelial keratoplasty (DMEK) as described previously [25]. Informed consent to corneal tissue donation and research use of tissue remnants had been obtained from the donors or their relatives. Experiments using human tissue samples were approved by the Institutional Review Board of the Medical Faculty of the University of Freiburg (25/20) and adhered to the tenets of the Declaration of Helsinki.

### Cell Isolation & Culturing

LEPC were isolated from limbal tissues of organ-cultured corneoscleral tissue (*n* = 210, mean age 66.3 ± 12.7 yrs; culture duration 22.7 ± 4.5 days; post-mortem time 30.6 ± 16.6 h) as previously published [26]. Briefly, limbal segments excised at 1 mm central and peripheral to the anatomical limbus were digested with collagenase A (Sigma-Aldrich; 2 mg/mL, 18 h, 37 °C). Cell clusters, isolated using 37 µm reversible strainers (Stem Cell Technologies, Köln, Germany), were dissociated with 0.25% Trypsin-EDTA. Single-cell suspensions from pooled tissues (4–6 corneae/preparation) were labeled CD117-PE and CD90-APC antibodies (5 µL/10⁶ cells) and DAPI (1:5000) for dead cell exclusion. FACS-sorted CD117⁻/CD90⁻ populations (LEPC) were cultured in keratinocyte serum-free medium (KSFM) complete medium (Keratinocyte serum-free basal medium + epidermal growth factor [5ng/ml] + bovine pituitary extract (BPE, 0.05mg/mL; Gibco, Grand Island, NY, USA)) at 37°C, 5% CO₂, with media changes every 48 h. LM (CD117^+^/CD90⁻) and LMSC (CD117⁻/CD90^+^) were cultured as previously described [24].

### Preparation of Conditioned Media and EV Isolation

LEPC (P1) were cultured in T75 flasks (four flasks/preparation) until reaching 70–80% confluence in KSFM as mentioned above. Subsequently, cell monolayers were washed twice with DPBS, and the KSFM complete medium was replaced with KSFM basal medium. Cells were cultured for 24 h to remove any leftover remnants of BPE and to adapt the cells to the basal medium. After 24 h, the medium was replenished and cells were cultured for an additional 72 h to generate a conditioned medium (CM). Unconditioned media served as negative controls. EV isolation was performed according to our previously published protocol [24]. Briefly, both CM and unconditioned media were collected and centrifuged at 2.500 g for 15 min at 4°C to remove any cells or cell debris. The resultant supernatant was subjected to sequential filtration through 0.8µm and 0.2 µm filters to eliminate large EVs (> 200 nm). The filtered media were subsequently concentrated to 1 mL using TFF (TFF-Easy; Hansa Biomed, Tallinn, Estonia), followed by 3 × PBS washes. Size-exclusion chromatography was performed using a qEVoriginal/35nm Gen 2 column (IZON Science, Christchurch, New Zealand) with 0.2 µm – filtered DPBS (Invitrogen, Waltham, MA, USA) as the mobile phase. The concentrated sample was loaded twice (500 µl each time). Following elution of 2.0 mL void volume (V0), eight sequential fractions (F1–F8, 500 µL each) were collected and stored at -80C until further use.

### Characterization of LEPC sEV

#### Measurement of Particle Count and Total Protein Concentration

The particle count and the total protein concentration were measured as described previously [24]. Briefly, the size distribution curves and concentration of particles (particle count) present in LEPC sEV fractions (F1–F8) were determined by NTA using the ZetaView BASIC PMX-120 instrument (Particle Metrix GmbH, Inning am Ammersee, Germany) equipped with NTA 2.0 analysis software. Using 100 nm standard beads, the instrument was calibrated, and the following settings were used: positions—11, cycles—1, minimum size—5 nm, maximum size—150 nm, trace length—15 s, sensitivity—80%, shutter speed—75 ms, frame rate—30, and temperature 23–24°C. Diluted LMSC- and LM-sEV fractions (100- to 5000-fold) were loaded into the NTA instrument and the values were recorded. Background measurements were performed with filtered PBS, which revealed the absence of any particles.

Micro-BCA assays (Thermo Scientific, Waltham, MA, USA) were performed following the manufacturer’s protocol to determine the free protein concentration of various EV fractions (F1-F8). Then, EV purity was determined by the ratio of particle count determined by NTA and free protein concentration determined by micro-BCA.

#### Transmission Electron Microscopy (TEM)

Negative staining was performed as previously described [24] to characterize the LEPC-sEV enriched fraction (F3), with respective fractions of unconditioned media serving as controls. Briefly, the Formvar-carbon-coated EM grids were subjected to UV irradiation (10 min) for surface hydrophilization. The sEV preparations were fixed by combining equal volumes with 4% paraformaldehyde (PFA), and 5 μl of the resulting suspension was deposited onto the prepared grids (*n* = 3 grids/sample). Following ambient incubation (5 min), specimens were sequentially processed by inverting onto droplets of: PBS (20 µL), 1% glutaraldehyde (20 µL, 5 min), and seven successive MQ-H20 washes (20 µL, 2 min each). Then, the grids were transferred to 30 μl drops of 2% uranyl oxalate solution (pH 7) for 5 min in the dark and to 30 μl drops of methyl cellulose-uranyl acetate for 10 min on ice in the dark. Excess solution was removed with stainless steel loops and Whatman filter paper, followed by ambient air drying (5–10 min). The grids were then analyzed using an electron microscope at 80 kV.

For cryo TEM, a droplet of the sample (3 μL) was applied on a holey carbon grid (Quantifoil R 1.2/1.3 Cu 300 mesh; glow discharged for 25 s at 10 mA (Pelco EasiGlow)) which was then blotted and plunge-frozen into the precooled liquid ethane (Vitrobot Mark IV, 22°C, 100% humidity, 6 s, force 5). To increase the particle density on the grid, multiple rounds of sample application and blotting were performed before the sample was plunge frozen. By embedding the sample in a thin layer of vitreous ice, the structure of the vesicles is preserved and protected from radiation damage. Samples were imaged on a 300 kV Krios G4 Cryo TEM with Selectris Energy Filter and Falcon 4i direct electron detector. Standard parameters were: 105kx magnification, total dose 40 e-/Å^2^, and 3 µm defocus. The area of each EV was measured from the obtained high-resolution pictures with the software ImageJ.

#### Western Blot Analysis

WB analysis was performed as described previously [24]. Briefly, EV fractions (15µl, ~ 10 µg; F1-F8) or whole-cell lysates (10 µg in RIPA buffer, ThermoFisher Scientific, Waltham, MA, USA) were solubilized using 4X Laemmli buffer (Biorad, Feldkirchen, Germany) at 95 °C for 10 min and were separated by SDS-PAGE under reducing conditions. Proteins were detected using specific antibodies (Supplementary Table 1) and horseradish peroxidase-labeled anti-mouse or rabbit IgG secondary antibodies (Jackson ImmunoResearch), visualized using ECL plus reagent (GE Healthcare) and Fusion FX7 Edge system 18.05 ( fusion fx Imager/fusion software, Vilber Lourmat; https://www.vilber.com/fusion-fx/).

#### Proteomics

sEV samples (F3) were eluted in STRAP extraction buffer (5% SDS, 50mM triethyl ammonium bicarbonate (TEAB; Sigma, T7408), pH 7.5) at 95 °C for 10 min. Samples were centrifuged at 13000g for 8 min and the supernatant used in the following steps. Proteins were reduced using 5 mM tris (2-carboxyethyl) phosphine hydrochloride (TCEP) (Sigma; 75259) for 10 min at 95°C and alkylated using 10 mM 2-iodoacetamide (Sigma; I1149) for 20 min at room temperature in the dark. The following steps were performed using S-Trap micro filters (Protifi, Huntington, NY) following the manufacturer’s procedure. Briefly, first a final concentration of 1.2% phosphoric acid and then six volumes of binding buffer (90% methanol; 100 mM TEAB; pH 7.1) were added. After gentle mixing, the protein solution was loaded to an S-Trap filter and spun at 2000 rpm for 0.5–1 min. The filter was washed three times using 150 μL of binding buffer. Sequencing-grade trypsin (Promega, 1:25 enzyme:protein ratio) diluted in 20µl digestion buffer (50 mM TEAB) were added into the filter and digested at 47 °C for 1 h. To elute peptides, three step-wise buffers were applied: a) 40 μL 50 mM TEAB, b) 40µl 0.2% formic acid in H2O, and c) 50% acetonitrile and 0.2% formic acid in H2O. The peptide solution were combined and dried in a SpeedVac.

Peptides were analyzed with the Evosep One system (Evosep Biosystems, Odense, Denmark) coupled to an timsTOF fleX mass spectrometer (Bruker). 500 ng of peptides were loaded onto Evotips C18 trap columns (Evosep Biosystems, Odense, Denmark) according to the manufacturer’s protocol. Peptides were separated on an EV1137 performance column (15 cm × 150 µm, 1.5 µm, Evosep) using the standard implemented 30 SPD method with a gradient length of 44 min (buffer A: 0.1% v/v formic acid, dissolved in H2O; buffer B: 0.1% v/v formic acid, dissolved in acetonitrile). Over the time of the gradient, the concentration of acetonitrile gradually increased from 0 to 90% at a flow rate of 500 nl/min.

The timsTOF fleX mass spectrometer was operated in the DIA-PASEF mode. DIA MS/MS spectra were collected in the range in an m/z range from 100 to 1700. Ion mobility resolution was set to 0.60–1.60 V·s/cm over a ramp time of 100 ms and an accumulation time of 100 ms. The collision energy was programmed as a function of ion mobility, following a straight line from 20 eV for 1/K0 of 0.6 to 59 eV for 1/K0 of 1.6. The TIMS elution voltage was linearly calibrated to obtain 1/K0 ratios using three ions from the ESI-L TuningMix (Agilent) (m/z 622, 922, 1222).

#### Bioinfomatics

Raw data were analyzed with DIA-NN software (v. 1.9) [27]. A spectral library was predicted using a FASTA file containing the human protein sequences as of July 22th, 2023 (human-EBI-reference database, https://www.ebi.ac.uk/). The false discovery rate (FDR) was set to 1%. The search was performed allowing one missed cleavage and cysteine carbamidomethylation enabled as a fixed modification. Quantification was performed using the label-free quantification algorithm MaxLFQ, which calculates the protein quantities as ratios from all peptide intensities. Only unique peptides were used for quantification. The Human-EBI-reference database was downloaded from https://www.ebi.ac.uk/ on March 3rd 2022. Label-free quantification (LFQ) intensity was used to quantify the proteins and those lacking LFQ intensity were eliminated. Gene Ontology (GO) enrichment analysis was conducted using the clusterProfiler (4.8.3) package [28] in the R (4.3.1) statistical environment [29]. The enrichplot(1.20.3) [30], pathview (1.40.0) [31], ggplot2 (3.4.4) [32], ComplexHeatmaps (2.16.0) [33], and ggVennDiagram (1.2.3) packages [34] were used for data visualization.

#### Immunohistochemistry

Immunostaining of frozen sections was performed as described in our earlier publication [35].

#### Statistics

All assays were performed in at least four independent experiments. Statistical analyses were performed using the GraphPad Instat statistical package for windows (Version 6.0; Graphpad Software Inc., La Jolla, CA, USA) and all the data were presented as mean ± standard deviation (SD). The statistical significance (*p* value < 0.05) was determined with Wilcoxon signed-rank test or Mann–Whitney U test as appropriate.

## Results & Discussion

### Enrichment and Characterization of sEV

#### Cell Isolation

**Fig. 1:**
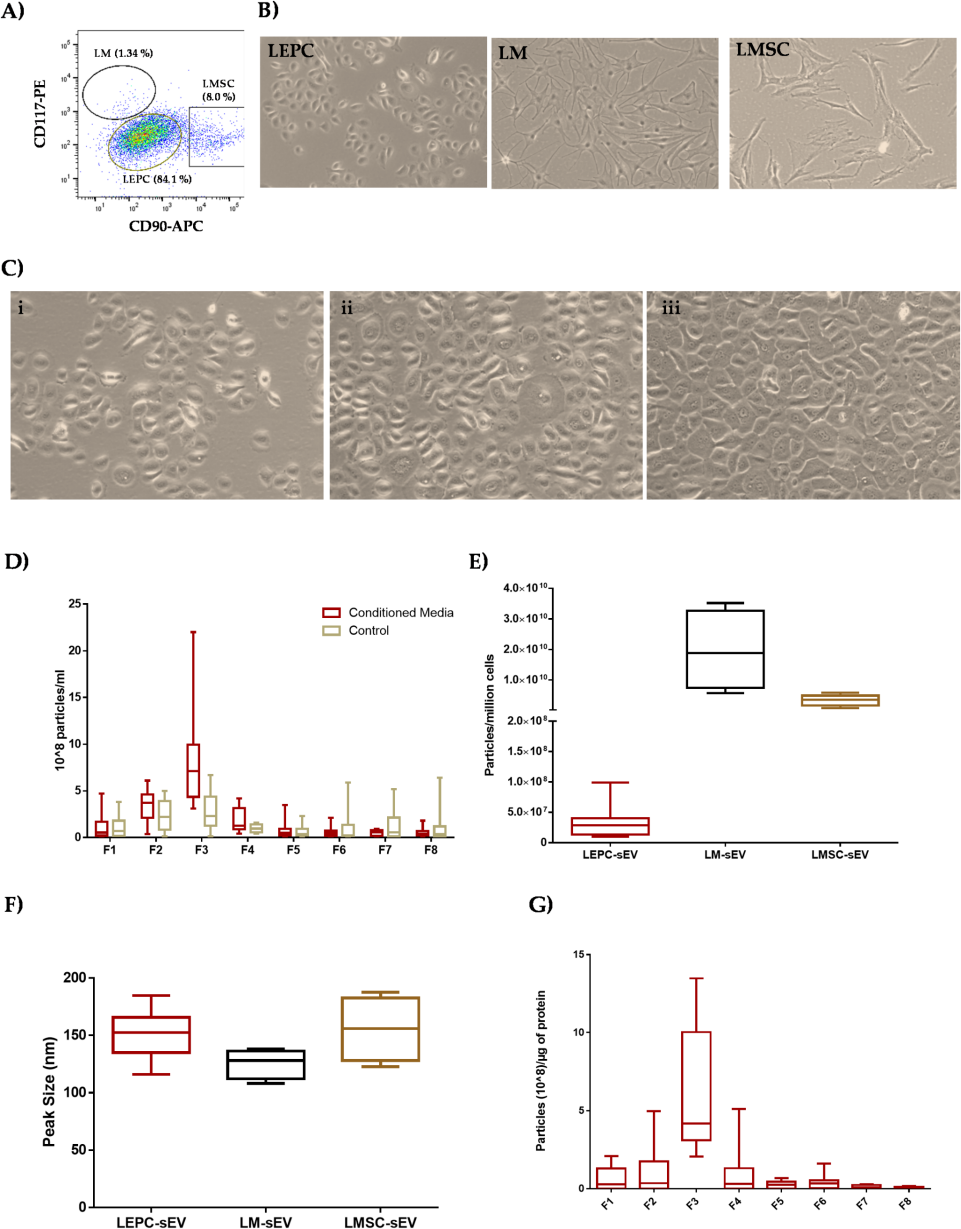
Enrichment and quantification of small extracellular vesicles (sEV): (**A**) Fluorescence-activated cell sorting (FACS) image showing the selection and separation of cells into CD90^+^CD117^−^ (LMSC), CD90^−^CD117^+^ (LM), and CD90^−^CD117^−^ (LEPC) populations. (**B**) Phase contrast images showing the morphological characteristics of the cell types: small cuboidal epithelial phenotype of limbal epithelial progenitor cells (LEPC); large, flattened, smooth bodies with multiple dendrites of limbal melanocytes (LM); spindle-shaped morphology with prominent nucleoli of limbal mesenchymal stromal cells (LMSC). **C**) Phase contrast images of LEPC before incubation in conditioned media (CM) (i), 24 h post incubation (ii), and on the day of CM harvesting (iii). **D**) Nanoparticle tracking analysis-showing particle counts in LEPC-sEV fractions (F1-F8) from conditioned and unconditioned media. Data presented as min to max whisker box plots (*n* = 14). **E**) The comparative analysis of particles produced/million cells of LEPC, LMSC and LM. Data presented as min to max whisker box plots (*n* = 14). **F**) Size distribution of LEPC-sEV and LM-sEV and LMSC-sEV particles in fraction 3 (F3) measured by nanoparticle tracking analysis. Data presented as min to max whisker box plots (*n* = 14/cell type). **G**) Particle-to-protein ratio analysis indicating the highest purity in fraction 3 (F3) for LEPC-sEV. Data are presented as min-to-max whisker box plots (*n* = 14)

Limbal cluster-derived cell suspensions from human donor corneas yielded of 1.6 ± 0.9% of CD117^+^ cells (LM, range: 405 – 9000 cells), 2.3 ± 1.1% of CD90^+^ cells (LMSC, range: 2000 – 31,600) and the remaining CD117^−^CD90^−^ cells (LEPC, range: 128,000 – 965,000) (Fig. 1A); gates were set based on isotype controls as described previously [26]. Primary cell cultures (Fig. 1B) exhibited cell-type-specific morphological phenotypes as described in our previous studies [24, 26].

#### sEV-enrichment

LEPC-sEV were produced using a KSFM basal medium devoid of animal components. The morphology of LEPC changed noticeably upon transitioning from KSFM complete medium to the KSFM basal medium. Initially, cells were sparsely distributed with predominantly rounded morphology and without any cell-cell connections (Fig. 1C-i). However, as they adapted to the basal medium, the cells became more confluent, increased in size and possibly developed intercellular connections (Fig. 1C-ii). By the time of sEV harvesting, the cells appeared in a tightly packed, cobblestone-like monolayer, indicative of full confluence (Fig. 1C-iii). After harvesting conditioned media, cell viability was evaluated using trypan blue exclusion staining, which demonstrated robust viability at 97.1 ± 2.1%. sEV were isolated from LMSC- and LM-conditioned media as described in our previous publication [24].

#### Biophysical Properties

LEPC-sEV were characterized by analyzing particle size and concentration (NTA), protein concentration (microBCA assay), morphology (electron microscopy), EV marker proteins (Western blot), and total protein cargo patterns (mass spectrometry).

#### Nant Particle Tracking Analysis

NTA revealed the highest particle concentration in fraction 3 (7.7 × 10^8^ (± 1.3) particles/mL) followed by fraction 2 (3.3 × 10^8^ (± 0.4)) and fraction 4 (1.5 × 10^8^ (± 0.3)) (Fig. 1D). Particle counts were significantly higher in the CM compared to the unconditioned medium, especially in fraction 3 (F3) (Fig. 1D). The absence of particles in the flow through (FT) or void volume (V) following TFF and SEC suggested complete particle retention. The particle counts observed in this study are consistent with previous reports for a human corneal epithelial cell line [36], despite differences in protocols and cell types. Interestingly, the number of particles of LEPC (3.3 × 10^7^ (± 2.5)/million cells) were much lower than that of the other major limbal cell types LMSC-sEV (3.3 × 10^9^ (± 1.8) particles/million cells) and LM-sEV (1.9 × 10^10^ (± 1.3) particles/million cells) (Fig. 1E). This data suggest cell-type specific differences in EV production rates. NTA analysis also revealed that LEPC-sEV had an average peak size at 150.5 ± 19.3nm (Fig. 1F), aligning with previous reports on corneal epithelial cell lines [23, 36] and primary human corneal epithelial cells [37]. No differences in particle size were observed between the cell types with LMSC-sEV averaging 155.6 ± 28.1nm, and LM-sEV 125.5 ± 12.8nm (Fig. 1F). Protein concentration analysis using the microBCA assay revealed the highest protein concentrations in F8 and flow through (FT) in both conditioned and unconditioned (control) media (Supplementary Fig. 1). F3 of LEPC-sEV exhibited the highest particle to protein ratio (6.1 × 10^8^ ± 4.1) (Fig. 1G), which was lower than previously reported for LMSC-sEV (3.4 × 10^9^ (± 1.6) but comparable to LM-sEV (7.1 × 10^8^ (± 2.0)) [24]. A higher particle-to-protein ratio indicates lower contamination and greater enrichment in EV preparations [38, 39], underscoring the efficiency of the isolation process and the high quality of EV preparations obtained.

#### Western Blot Analysis

**Fig. 2:**
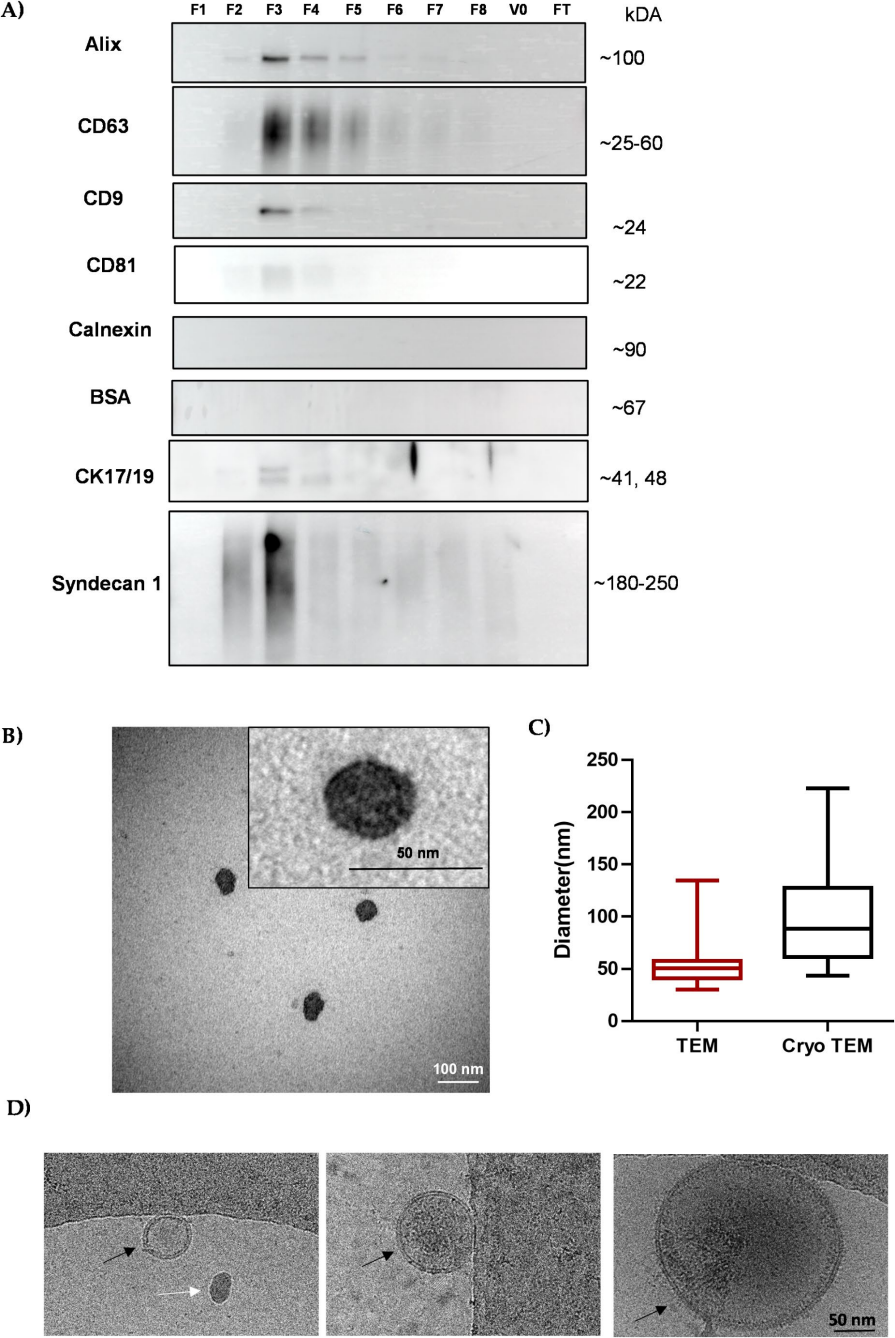
Characterization of small extracellular vesicles (sEV): (**A**) Western blot analysis of EV-specific markers in fractions F1-F8, void volume V0 and flow through FT from LEPC-sEV. Enrichment of EV markers (Alix, CD63, CD9, CD81) were predominantly observed in F3 fraction. The absence of the endoplasmic reticulum marker calnexin (CANX) and residual bovine serum albumin (BSA) across all fractions indicates a lack of cellular contamination and serum residue. Limbal epithelial progenitor markers CK17/19 and epithelial major syndecan, syndecan 1, were detected in F2-F4, with the highest levels in F3. Uncropped Western blot images are provided in Supplementary Figures – S2-S7. (**B**) Transmission electron microscopy (TEM) images of F3 fractions showing classical EV morphology. (**C**) Size distribution of LEPC-sEV in F3 based on TEM and Cryo TEM analysis. Data presented as min to max whisker box plots (*n* = 3). **D**) Cryo TEM images of F3 fraction showing sEV of varying sizes with classical EV morphology (black arrows) and intact membranes. White arrow showing an ice contamination

WB analysis was performed to characterize the different fractions, focusing on transmembrane proteins (CD9, CD63, CD81), the cytosolic EV protein Alix (PDCDIP), cell type-specific marker (CK17) [40], and the non-EV marker calnexin (CANX) and contaminant bovine serum albumin (BSA) [38]. WB analysis revealed that Alix was predominantly enriched in F3 with a stronger band observed (Fig. 2A & Supplementary Fig. 2). Similarly, CD9, CD63 and CD81 were present in fractions 2–5 but predominantly found in F3 (Fig. 2A). This distribution was similar to our previously published data on LMSC-sEV and LM-sEV [24], except for CD81 expression, which showed only a faint band in F3 of LEPC-sEV. This correlated with the lower CD81 expression levels in LEPC lysates compared to LMSC and LM cell lysates (Supplementary Fig. 3). Importantly, the absence of the endoplasmic reticulum-resident protein CANX across all LEPC-sEV fractions (Fig. 2A), but its presence in the cell lysates (Supplementary Fig. 4), and BSA (Fig. 2A & Supplementary Fig. 5), confirmed the effective isolation of sEV without contamination from intracellular components and any serum components [38]. The LEPC-specific marker CK17/19 and epithelial major syndecan, syndecan 1 was detected in the fractions 2–4, with the strongest signal in F3 (Fig. 2A & Supplementary Fig. 6 & 7). In contrast, no EV- or cell-specific markers were detected in unconditioned media. Based on these findings, which confirmed the enrichment of sEV in F3, this fraction was used in subsequent experiments.

#### Electron Microscopy

TEM analysis revealed that the particles in F3 displayed the characteristic morphology of EVs, with an intact membrane and a doughnut or cup-shaped structure (Fig. 2B). The LEPC-sEV had a size of 52.4 ± 15.5 nm in negative stain TEM (Fig. 2C), similar to previous studies on LEPC exosomes [22, 41]. The size distribution was also similar to LMSC- and LM-sEV particles in our earlier study [24]. NTA measurements revealed larger EV diameters than TEM, consistent with previous findings [24, 42, 43] likely due to EV shrinkage with formaldehyde fixation during TEM preparation.

Cryo TEM analysis of LEPC-sEV revealed that the majority of the particles were spherical, electron-dense, and exhibited a well-defined lipid bilayer (Fig. 2D). The size distribution of the vesicles ranges from approximately 50 nm to 200 nm (98.8 ± 44.6 nm, Fig. 2C & D), highlighting heterogeneity in their morphology and dimensions.

#### Proteomics Analysis

**Fig. 3:**
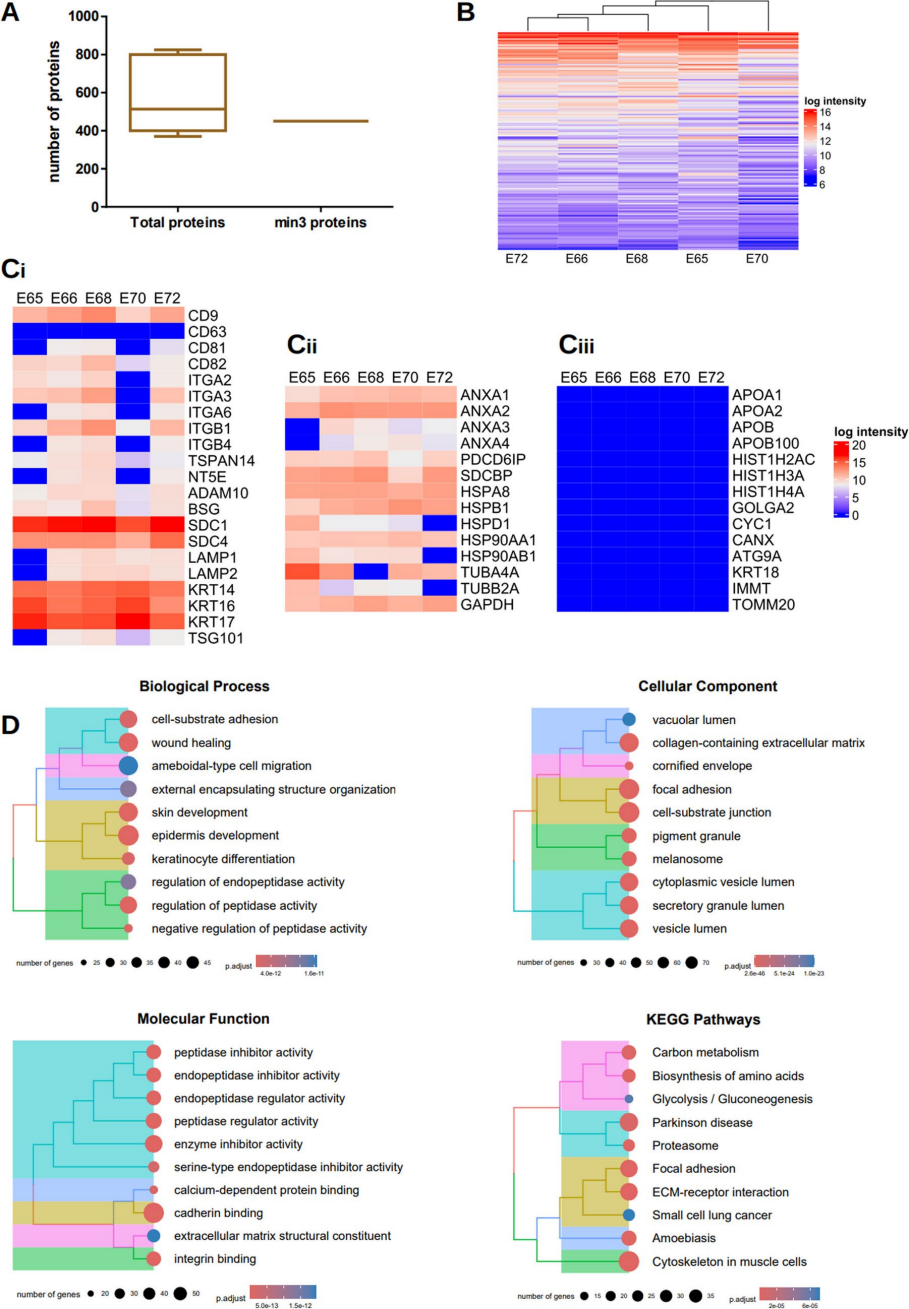
Proteomic characterization of small extracellular vesicles (sEV): (**A**) Box plot showing the number of proteins identified in LEPC-sEV (*n* = 5) and the number of proteins commonly detected in at least three samples. **B**) Heatmap displaying unbiased clustering of protein intensity profiles from different biological samples based on LFQ intensities. **C**) Heatmaps showing the protein intensity profiles of (i) plasma membrane, endosomal and cell-specific proteins; (ii) cytosolic proteins; (iii) apolipoproteins and cellular contaminants in LEPC-sEV samples. (**D**) Top 10 enriched Gene Ontology (GO) terms for Biological Process, Molecular Function, Cellular Component and KEGG pathways in LEPC-sEV

To establish an unbiased protein inventory of LEPC-derived sEV, we performed quantitative proteomic profiling mass spectrometry (*n* = 5). The analysis identified 1307 unique proteins based on LFQ intensity, of which 450 proteins were consistently detected in ≥ 3 biological replicates per group (Fig. 3A), indicating the inherent variability among the samples. Unsupervised hierarchical clustering of the samples, as shown in the heatmaps (Fig. 3B), revealed distinct patterns of protein expression. We first characterized the general proteome of LEPC-sEV, then conducted comparative analyses with previously reported LMSC-sEV and LM-sEV proteomics data [24] to identify both LEPC-specific signature proteins and shared signatures across all limbal cell type-derived sEV.

#### General sEV Proteome

The proteomics datasets revealed a repertoire of proteins (identified by official gene symbols) associated with the plasma membrane (PM) and/or endosomes in LEPC-sEV. These included tetraspanins (CD9, CD81, CD82, TSPAN14), 5’ nucleotidase (NTE5), integrins (ITG) (α2, α3, β1, β4), multipass membrane proteins (BSG, ADAM10), a complement binding protein (CD59), syndecans (SDC1, SDC4), HLA-class A, lysosome-associated membrane glycoprotein (LAMP1/2) (Fig. 3C-i). Cytosolic proteins associated with endosomal sorting complexes required for transport (ESCRT)-1 complex protein (TSG101), ALIX (PDCD6IP), annexins (A1, A2, A3, A4), syntenin (SDCBP), and heat shock proteins (HSP90AA1, HSP90AB1, HSPA8) (Fig. 3C-ii), were also detected. These proteins are known to play roles in EV formation, recipient cell binding, and membrane fusion [38]. The proteomics findings were corroborated by WB analysis, which confirmed the presence of key EV markers CD9, CD81 and Alix (Fig. 2A). The detection of heat shock proteins was consistent with a previous study on a corneal epithelial cell line [41]. Interestingly, while CD63 was absent in the proteomics data, it was detected in all samples by WB. This discrepancy likely stems from differences in detection sensitivity and specificity between techniques. Proteins like CD63 might have been underrepresented in the proteomics data due to low abundance, poor ionization, or incomplete peptide coverage. Furthermore, EV contained known lineage-specific antigens [26], such as keratins (KRT14, KRT17) indicating the enrichment of distinct vesicle signatures generated by LEPC (Fig. 3C-i). The detection of KRT17 (CK17) and syndecan 1 in LEPC-sEV aligned with WB data (Fig. 2A). Along with previous studies [44, 45], tubulin (TUBA4A, TUBB2A), and the enzyme (GAPDH) were identified in LEPC-sEV (Fig. 3C-ii). These findings suggest that, while common vesicle-associated proteins were present, specialized integrins, receptors, and lineage antigens unique to LEPC are incorporated in sEV. This highlights a discrete sEV cargo composition, aligning with the differential functional activities of LEPC**.**

#### Assessment of sEV Purity

To evaluate sEV purity, it is essential to quantify common contaminants such as apolipoproteins, transmembrane proteins, lipid-bound proteins, and soluble proteins associated to other intracellular compartments [38]. Analysis revealed that apolipoproteins A1/2 (APOA1/2), B (APOB), APOB100, and bovine serum albumin (BSA) were absent in LEPC-sEV (Fig. 3C-iii). Importantly, LEPC-sEV lacked markers associated with the nucleus (e.g., histones (HIST1H)), mitochondria (IMMT, CYC1, TOMM20), endoplasmic reticulum (CANX), Golgi apparatus (GOLGA2), or autophagosomes (ATG9A) as well as cytoskeletal KRT18 (Fig. 3C-iii), distinguishing them from debris such as apoptotic bodies or shed membrane components [38, 46]. Notably, the absence of CANX in LEPC-sEV was corroborated by WB analysis (Fig. 2A). These findings suggest that the LEPC-sEV preparation is highly pure, minimizing contamination from non-sEV components.

#### Functional Enrichment Analysis

Gene ontology (GO) and Kyoto Encyclopedia of Genes and Genomes (KEGG) pathway enrichment analysis of the sEV proteomic data sets were performed in R using appropriate software packages to elucidate associated biological processes (BP), molecular functions (MF), cellular components (CC), and signaling pathways (Supplementary Table 2). The top 10 enriched BP, CC, MF and KEGG pathways, ranked by significance, are shown in Fig. 3D. Proteomic analysis revealed that LEPC-sEV are enriched in proteins involved in wound healing, keratinocyte differentiation, epidermis development; regulation of peptidation activity and extracellular matrix organization (Fig. 3D). Pathway analysis highlighted the roles of LM-sEV in focal adhesion and ECM-receptor interactions (Fig. 3D). Notably, LEPC-sEV appear to contribute specifically to epithelial differentiation and development, processes critical for maintaining tissue integrity and function. These data suggest that LEPC-sEV, alongside our previously published LMSC- and LM-sEV play distinctive roles in regulating cell-matrix interactions, cell adhesion signaling and cytoskeletal dynamics. This may highlight their crucial involvement in intercellular communication and sensing of the microenvironment cues.

#### Comparative sEV Proteome Profiling

**Fig. 4:**
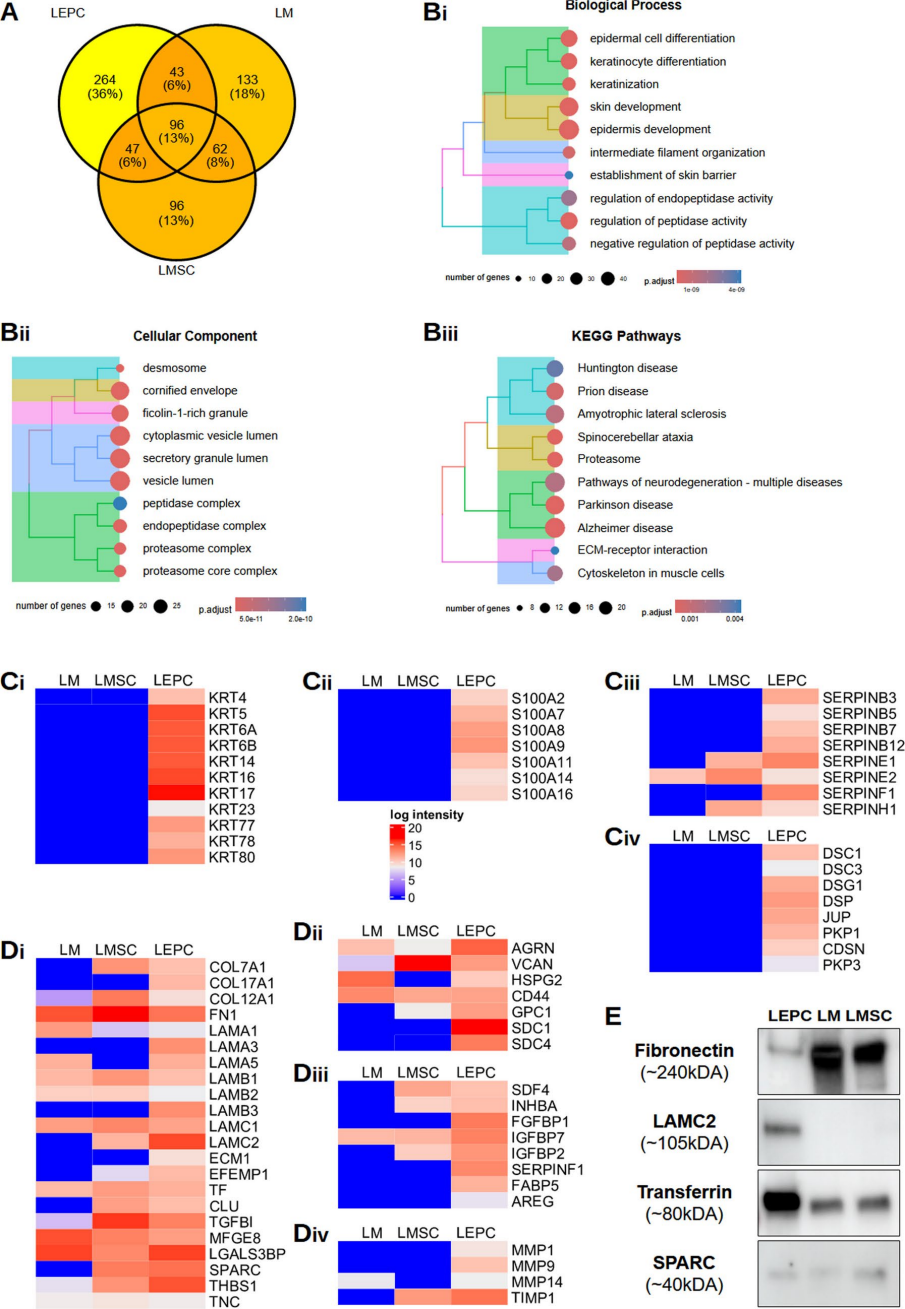
Comparative proteomic profiling of limbal epithelial progenitor cell derived small extracellular vesicles (sEV), limbal melanocyte (LM)-sEV and limbal mesenchymal stromal cell (LMSC) derived –sEV. (**A**) Venn diagram showing the number of unique and shared proteins identified among LEPC-sEV, LM-sEV and LMSC-sEV. **B**) Gene ontology analysis highlighting the unique biological processes, cellular components, molecular functions, and KEGG pathways enriched in proteins found exclusively in LEPC-sEV. (**B**) Heatmaps showing selected protein clusters uniquely present in LEPC-sEV, categorized as follows keratin profiling (i), s100 calcium binding proteins (ii), serpin family proteins (iii) and desmosomal related family proteins (iv). **C**) Heatmaps showing the selected ECM-related proteins including collagens, laminins and other glycoproteins (i), proteoglycans (ii), growth factors (iii) and tissue remodeling proteins. (**C**) Western blot validation showing: Fibronectin (FN1) in all three populations, with predominant expression in LM-and LMSC-sEV samples; SPARC in all three populations; transferrin predominantly expressed in LEPC-sEV; LAMC2 restricted to LEPC-sEV populations. Uncropped versions of the Western blot are shown in Supplementary Fig. 8

To identify LEPC-specific signature proteins and shared signatures across limbal cell type-derived sEV, we compared the LEPC-sEV proteome data with our previously reported LMSC-sEV and LM-sEV proteome data [24]. The Venn diagram demonstrates that 13.0% of proteins were found in all cell type derived sEV; 6% proteins were shared between LEPC-and LM-sEV as well as LEPC and LMSC-sEV, whereas 36% of proteins were exclusively present in LEPC sEV (Fig. 4A). The proteins commonly found among all cell types related to the classical EV-related markers and basic ECM constituents (Supplementary Table 3).

Many of the proteins found exclusively in LEPC-sEV are related to epidermis development, epidermal/keratinocyte differentiation, intermediate filament organization and also were involved in the regulation of peptidase activity (Fig. 4B-i&ii, Supplementary Table 4), in keeping with the specialized function of the parent epithelial cells. KEGG pathway analysis highlighted connections of LEPC-sEV to proteasome, cytoskeleton, and pathways of neurodegeneration (Fig. 4B-iii), Supplementary Table 4).

#### Structural and Regulatory Protein Families

The predominant exclusive protein families identified in LEPC-sEV comprised keratins, S100 proteins, serpins, and desmosomal-complex proteins. These clusters represent functionally distinct protein families with significant roles in epithelial biology.

##### Keratins

Keratins are essential structural components of intermediate filaments that provide mechanical support and facilitate the formation of desmosomes between cells and hemidesmosomes with basement membranes, thereby maintaining epithelial integrity. While keratins are highly abundant cellular cytoskeletal proteins frequently observed in sEV and often considered contaminants [47], analysis of LEPC-sEV revealed enrichment of multiple keratin subtypes. These included type I keratins (KRT14, KRT16, KRT17, KRT23) and type II keratins KRT4, KRT5, KRT6A, KRT6B, KRT77, KRT78 and KRT80 (Fig. 4C-i). This keratin profile aligns with previous reports characterizing the limbal epithelium [48–51] and suggests that the secreted vesicles preserve cell-type specific characteristics. Notably, KRT17, an established marker for LEPC identification [40], aligned with Western blot analysis (Fig. 2A). Interestingly, in a recent proteomic study of EVs derived from a corneal epithelial cell line, keratins KRT18, KRT19, KRT7, and KRT8 were enriched [23]. The absence of these proteins in our proteomics analysis highlights differences in the cellular origin of the vesicles (primary cells vs. cell lines). Additionally, KRT18, often associated with oncosomes, and keratin 10, a skin keratin known to appear as a contaminant in mass spectrometry analyses [47], were absent in LEPC-sEV, further supporting the purity and specificity of our preparations.

##### S100 Proteins

The S100 calcium-binding protein family, which regulates various biological processes through calcium-dependent interactions with target proteins demonstrated significant enrichment in LEPC-sEV (Fig. 4C-ii). This observation is consistent with recent findings documenting S100 proteins in EV-DNA-associated proteins complexes [52, 53]. The spatial distribution of S100 proteins in the limbal region was documented with S100A2 expression in basal limbal epithelial cells, while S100A8 and S100A9 were found in suprabasal/superficial limbal epithelial cells. S100A8 and S100A9 showed elevated expression levels in inflammatory ocular conditions, including dry eye syndrome and pterygium [54], suggesting their possible involvement in mediating inflammatory responses in these pathological conditions.

##### Serpins

Serine protease inhibitors (SERPIN), a significant protein family in our analysis, act as critical molecular switches in ECM remodeling cascades, influencing both immune cell recruitment and re-epithelization through a combination of direct contact and indirect intercellular signaling mechanisms. The presence of serpins on EV have been previously documented, with studies demonstrating that serpin-loaded EVs promote tissue repair in mouse models [55]. Our proteomic analysis identified various serpin family proteins in LEPC-sEV (Fig. 4C-iii), including SERPINB3, B5, B7, B12, F1, which plays a crucial role in maintaining corneal epithelial homeostasis [56, 57]. Notably, these serpins were exclusively present in LEPC-sEV. In contrast, SERPINH1 and E1 were detected in both LMSC- and LM-sEV, while SERPINE2 was shared among sEV from three cell types (Fig. 4C-iii).

##### Desmosomal Complex Proteins

The LEPC-sEV uniquely contained desmosomal complex proteins including desmogelins (DSG1), desmocolins (DSC1 and 3), plakophilins (PKP1 and 3), desmoplakins (DSP), plakoglobin (JUP) (Fig. 4C-iv), similar to previous findings in retinal pigmented epithelium -EV [58]. These adhesive intercellular junction proteins are characteristically present throughout the corneal epithelial cell layers, particularly between interdigitating cell borders of wing cells.

#### ECM Matrix Proteins and Niche Regulation

The limbal niche ECM, composed of collagens, glycoproteins and proteoglycans, provides structural support and enables cell communication [4, 59, 60]. Recent studies indicate that EV serve as ECM components, contributing to matrix organization, and regulation of resident cells across various connective tissues [61, 62]. Our proteomic analysis revealed distinct patterns of ECM protein distribution among different cell-derived sEV. Collagen type XVII (COL17A1) was exclusively present in the LEPC-sEV, whereas collagens COLVII (COL7A1) and COLXII (COL12A1) were present in sEV from all three cell types (Fig. 4D-i). The presence of COL17 aligns with previous reports documenting its expression in adult human cornea and limbus [4]. Our analysis further revealed multiple laminin (LN) subunit distributions across the vesicle populations. The β1(LAMB1), and γ1 (LAMC1) chains were identified in sEV from all three cell types (Fig. 4D-i). Notably, the limbal specific LN-α5 (LAMA5), which forms LN10/11 and promotes the growth of LEPC and LM [63, 64], was detected in both LM-sEV and in LEPC-sEV (Fig. 4D-i). Laminin-332, a crucial basement protein of ocular surface epithelium that supports LEPC adhesion and migration [64], showed a distinct distribution pattern. The α3 and β1 chains were exclusively present in the LEPC-sEV, while (LAMC2) was found in both LEPC- and LMSC-sEV (Fig. 4D-i). WB analysis confirmed that γ2 expression was restricted to LEPC (Fig. 4E), consistent with our previous RNA expression studies [63], suggesting LEPC-mediated secretion of LN-332 via EV for basement membrane deposition.

Several ECM glycoproteins were present in LEPC-sEV populations, including fibronectin (FN1), thrombospondin 1 (THBS1), transforming growth factor β-inducible (TGFBI), milk fat globule-EGF factor 8 (MFGE8) and galectin-3-binding protein (LGASL3BP), Transferrin (TF), and SPARC (Fig. 4D-i), similar to previous reports on a corneal epithelial cell line [41]. Western blot analysis confirmed TF, FN1, SPARC expression across all three cell type sEV, with TF rendering a strong band in LEPC-sEV, SPARC in LMSC-sEV, whereas FN was detected in in both, LMSC-sEV and LM-sEV (Fig. 4E). LEPC-sEV exclusively contained extracellular matrix protein 1 (ECM1), EGF-containing fibulin extracellular matrix protein (EFEMP1, also called ocular fibulin 3) (Fig. 4D-i). The proteoglycan agrin (AGRN) was found in sEV of all cell types, whereas versican (VCAN) as well as BM- specific perlecan (HSPG2) were detected in both, LEPC- and LM-sEV (Fig. 4D-ii). The cell-surface proteoglycan CD44 was found in sEV from all three cell types, whereas glypican 1 was present in LEPC- and LMSC-sEV (Fig. 4D-ii). These distinct ECM protein signatures align with the specialized niche microenvironments and progenitor cell regulation of these cell types.

#### Growth Factors, Their Binding Proteins and Matrix-remodelling Proteins

Accumulating evidence indicates that growth factors and soluble mediators are associated with the EV surface, implicating EV in the systemic dissemination of bioactive growth factors [65]. Differential enrichment analysis revealed distinct growth factors and bioactive mediators, such as fibroblast growth factor binding protein (FGBP1), Insulin growth factor binding proteins 2 and 7, fatty acid binding protein 5 (FABP5) and amphiregulin (AREG) as exclusively present in LEPC-sEV (Fig. 4D-iii). SDF4 and inhibin subunit β (INHBA) were detected in both, LEPC-sEV and LMSC-sEV (Fig. 4D-iii). The identified IGFBP2 and 7 (Fig. 4D-iii) play roles in angiogenesis, with IGFBP7 serving as a transforming growth factor –β1 target [66], and a biomarker of conjunctivalization in limbal stem cell deficiency [67]. The growth factor profile of LEPC-sEV differs from pevious findings in corneal epithelial cell line proteomics, likely due to variations in protocols used for EV isolation and cell types used [41]. Matrix remodeling enzymes were also identified as sEV cargo. Matrix metalloproteinases (MMP1, MMP9, MMP14) were exclusively detected in LEPC-sEV, while tissue inhibitors of metalloproteinase (TIMP1) was present in both LMSC- and LEPC-sEV (Fig. 4D-iv). These enzymes, crucial for wound re-epithelialization through ECM degradation and deposition during tissue injury [68], suggest potential sEV-involvement in limbal niche turnover [61, 69].

## Conclusions

This study successfully isolated sEV from a major key cell type in the limbal stem cell niche, LEPC. Proteomic analysis revealed that these vesicles may play signaling roles related to epidermal cell differentiation/development, ECM regulation and intercellular communication within the niche microenvironment as they carry distinct subsets of ECM molecules and proteins involved in ECM modulation. Further analysis is needed to elaborate the specific functional relevance of the isolated sEV in mediating limbal niche homeostasis.

### Limitations of the Study

While this study provides valuable insights into the distinct sEV populations derived from LEPC through comprehensive characterization techniques, some limitations warrant consideration. First, the reliance on in vitro cultured cells raise concerns about the extent to which the observed sEV profiles accurately represent limbal niche environment in vivo. Additionally, the use of organ-cultured corneas for cell isolation, rather than fresh limbal tissues may have influenced the cellular properties and, by extension, the characteristics of secreted sEV. Another notable limitation, the study lacked functional assays to directly demonstrate the role of LEPC-sEV in regulating limbal niche homeostasis or epithelial regeneration, limiting the functional implications that can be drawn. Future studies addressing these limitations, including the incorporation of in vivo models and functional assays, could provide a more comprehensive understanding of the specialized roles of LEPC-sEV within in the limbal stem cell microenvironment and their potential contributions to tissue homeostasis and regeneration.

## Supplementary Information

Below is the link to the electronic supplementary material.

Supplementary file1 (PPTX 15577 KB)

Supplementary file2 (DOCX 19 KB)

Supplementary file3 (XLSX 351 KB)

Supplementary file4 (XLSX 10 KB)

Supplementary file5 (XLSX 103 KB)

## Data Availability

Data will be made available on reasonable request.
