# Supplementary material for: Proteomic Insights into Human Limbal Epithelial Progenitor-Derived Small Extracellular Vesicles
Source: Stem Cell Rev Rep. 2025 Apr 16;21(5):1578–93. doi: 10.1007/s12015-025-10877-w (PMC12316787; doi:10.1007/s12015-025-10877-w)
Supplement: Supplementary file 1 — Supplementary file1 (PPTX 15577 KB) [file 12015_2025_10877_MOESM1_ESM.pptx]

## Slide 1
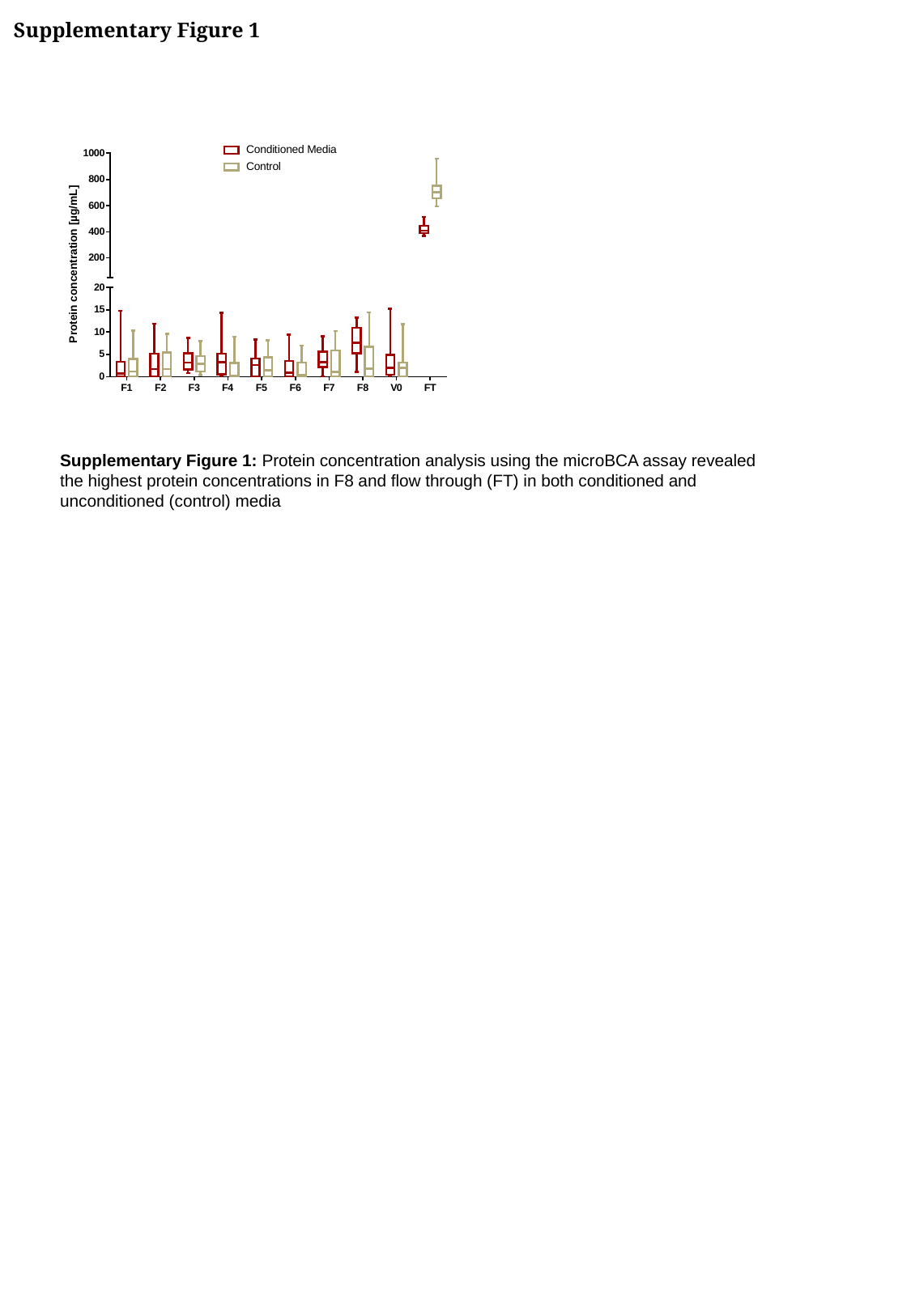

Supplementary Figure 1
Supplementary Figure 1: Protein concentration analysis using the microBCA assay revealed the highest protein concentrations in F8 and flow through (FT) in both conditioned and unconditioned (control) media

## Slide 2
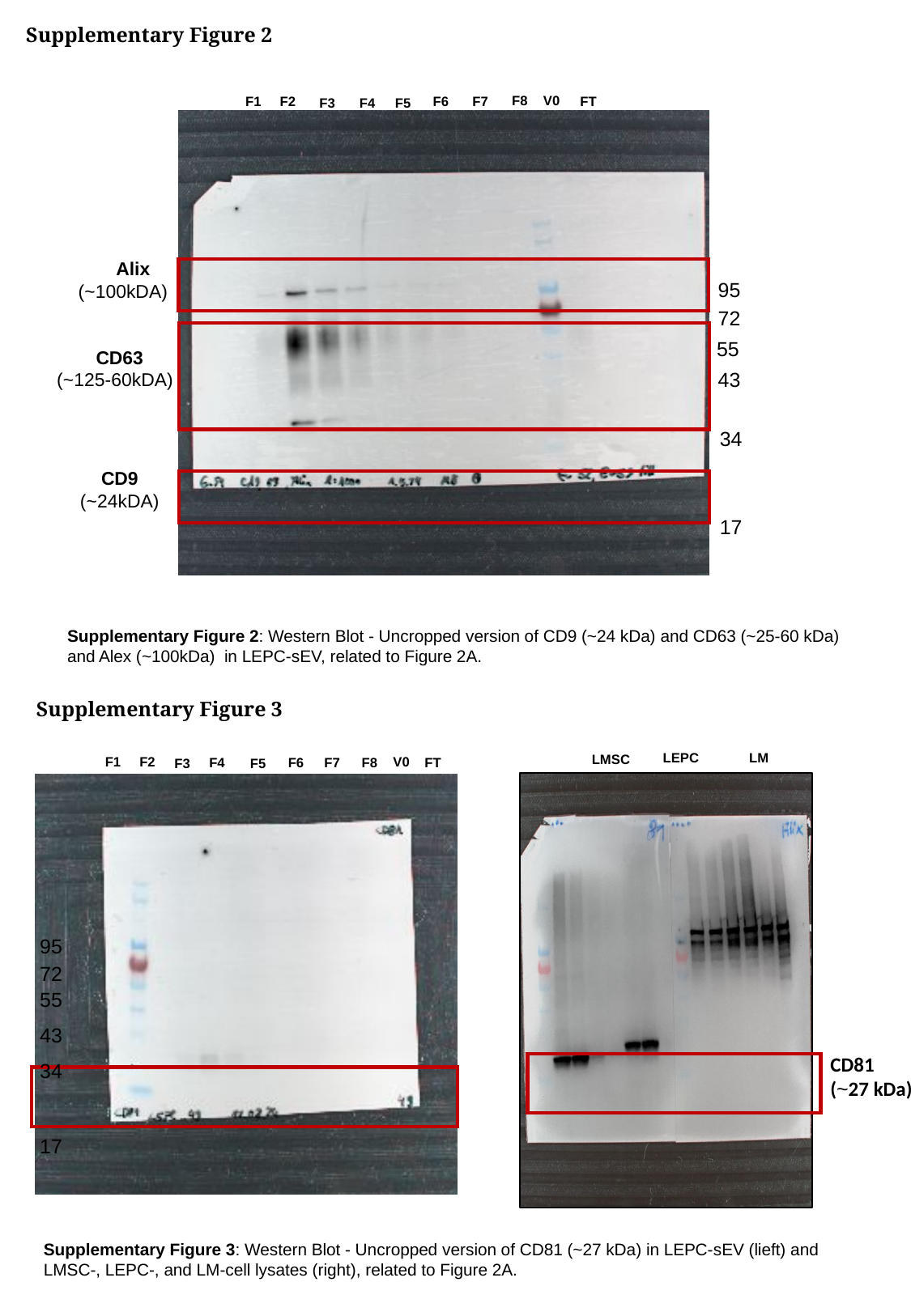

Supplementary Figure 2
V0
F8
FT
F1
F2
F6
F7
F3
F4
F5
Alix
(~100kDA)
95
72
55
43
34
17
CD63
(~125-60kDA)
CD9
(~24kDA)
Supplementary Figure 2: Western Blot - Uncropped version of CD9 (~24 kDa) and CD63 (~25-60 kDa) and Alex (~100kDa) in LEPC-sEV, related to Figure 2A.
Supplementary Figure 3
LEPC
LM
LMSC
F1
V0
F2
F4
F6
F7
F8
FT
F3
F5
95
72
55
43
34
17
CD81
(~27 kDa)
Supplementary Figure 3: Western Blot - Uncropped version of CD81 (~27 kDa) in LEPC-sEV (lieft) and LMSC-, LEPC-, and LM-cell lysates (right), related to Figure 2A.

## Slide 3
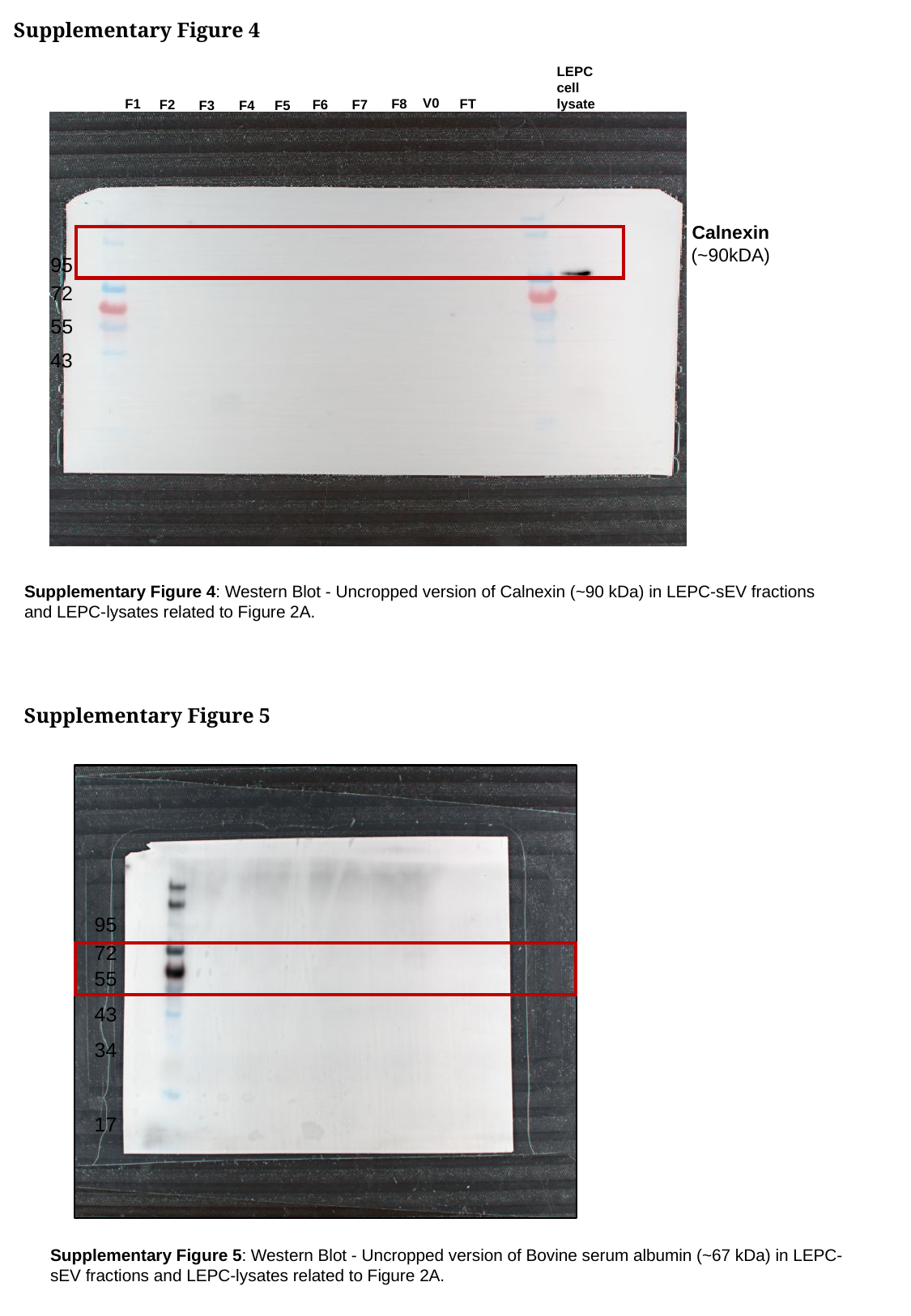

Supplementary Figure 4
LEPC cell lysate
V0
F8
FT
F1
F2
F6
F7
F3
F4
F5
Calnexin
(~90kDA)
95
72
55
43
Supplementary Figure 4: Western Blot - Uncropped version of Calnexin (~90 kDa) in LEPC-sEV fractions and LEPC-lysates related to Figure 2A.
Supplementary Figure 5
95
72
55
43
34
17
Supplementary Figure 5: Western Blot - Uncropped version of Bovine serum albumin (~67 kDa) in LEPC-sEV fractions and LEPC-lysates related to Figure 2A.

## Slide 4
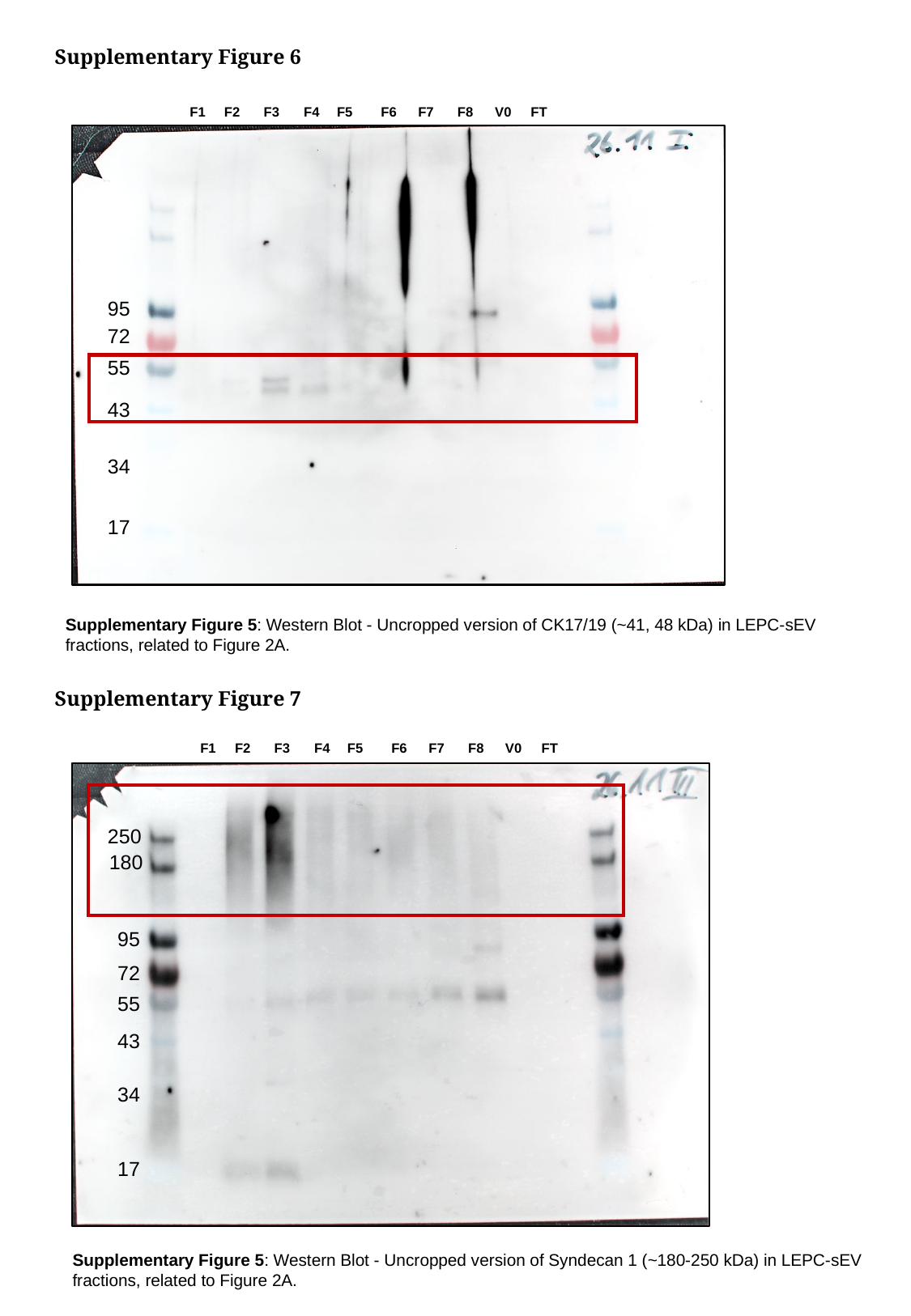

Supplementary Figure 6
F1
F2
F3
F4
F5
F6
F7
F8
V0
FT
95
72
55
43
34
17
Supplementary Figure 5: Western Blot - Uncropped version of CK17/19 (~41, 48 kDa) in LEPC-sEV fractions, related to Figure 2A.
Supplementary Figure 7
F1
F2
F3
F4
F5
F6
F7
F8
V0
FT
250
180
95
72
55
43
34
17
Supplementary Figure 5: Western Blot - Uncropped version of Syndecan 1 (~180-250 kDa) in LEPC-sEV fractions, related to Figure 2A.

## Slide 5
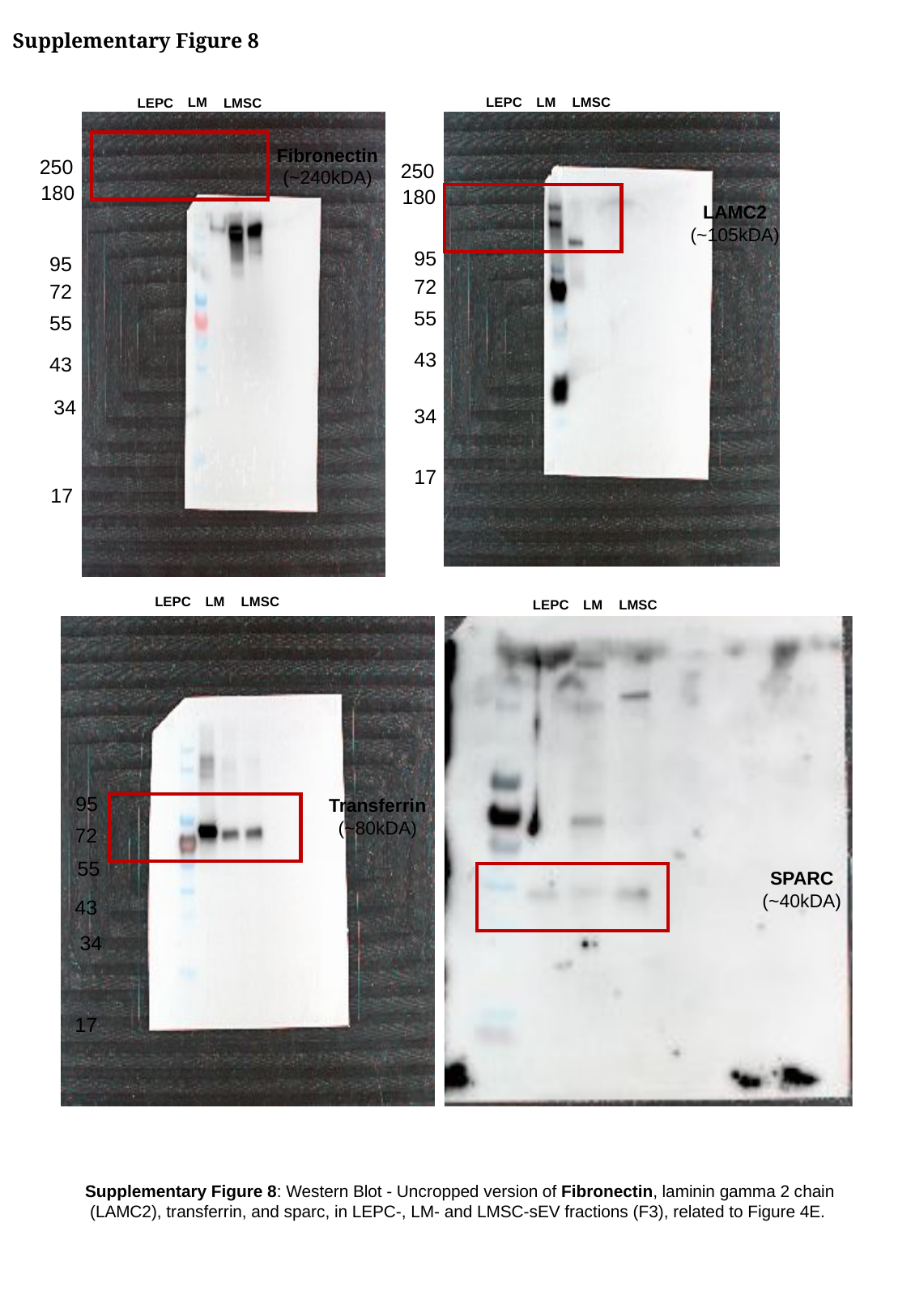

Supplementary Figure 8
LM
LEPC
LMSC
LM
LEPC
LMSC
Fibronectin
(~240kDA)
250
250
180
180
LAMC2
(~105kDA)
95
72
55
43
34
17
95
72
55
43
34
17
LM
LEPC
LMSC
LM
LEPC
LMSC
95
72
55
43
34
17
Transferrin
(~80kDA)
SPARC
(~40kDA)
Supplementary Figure 8: Western Blot - Uncropped version of Fibronectin, laminin gamma 2 chain (LAMC2), transferrin, and sparc, in LEPC-, LM- and LMSC-sEV fractions (F3), related to Figure 4E.
