# Supplementary material for: Proteomic Insights into Human Limbal Epithelial Progenitor-Derived Small Extracellular Vesicles
Source: Stem Cell Rev Rep. 2025 Apr 16;21(5):1578–93. doi: 10.1007/s12015-025-10877-w (PMC12316787; doi:10.1007/s12015-025-10877-w)
Supplement: Supplementary file 2 — Supplementary file2 (DOCX 19 KB) [file 12015_2025_10877_MOESM2_ESM.docx]

**Supplementary Table 1: List of antibodies used in the study**

| **Antibody (clone), Host species** | **Antibody dilution** | **Application** | **Antibody source** | **Catalog number** |
| --- | --- | --- | --- | --- |
| Alix (E6P9B) Rabbit mAb | 1/1000 | Western Blot | Cell Signaling Technology | 92880S |
| CD63 (E1W3T) Rabbit mAb | 1/1000 | Western Blot | Cell Signaling Technology | 52090S |
| CD9 (D3H4P) Rabbit mAb | 1/1000 | Western Blot | Cell Signaling Technology | 13403S |
| CD81 (D3N2D) Rabbit mAb | 1/1000 | Western Blot | Cell Signaling Technology | 56039S |
| Melan-A (EPR20380), rabbit mAb | 1/1000 | Western Blot | Abcam | ab210546 |
| CD90/Thy1 (7E1B11), mouse | 1/500 | Western Blot | Novus Bio | NBP2-37330 |
| Calnexin (C569) Rabbit mAb | 1/1000 | Western Blot | Cell Signaling Technology | 2679S |
| CK17/19 (D4G2)  Rabbit mAb | 1/1000 | Western Blot | Cell Signaling Technology | 12434 |
| BSA (D108Q) Rabbit mAb | 1/1000 | Western Blot | Cell Signaling Technology | 23053S |
| Fibronectin (IST-4) mouse | 1/250 | Western Blot | Merck/ Sigma Aldrich | F0916 |
| SPARC, Rabbit | 1/500 | Western Blot | Invitrogen | PA5-78178 |
| CD117, PE (YB5.B8), Mouse | 5µl/10^6^ cells | Flow Cytometry | BD Biosciences | 561682 |
| CD90, APC (5E10), Mouse | 5µl/10^6^ cells | Flow Cytometry | BD Biosciences | 559869 |
| LAMC2 (E9F7M), Rabbit mAB | 1/1000 | Western blot | Cell Signaling Technology | 53884 |
| Anti-Mouse IgG, Horseradish Peroxidase (Polyclonal), Goat | 1/10000 | Western Blot | Jackson ImmunoResearch Labs | 115-035-003 |
| Anti-Rabbit IgG, Horseradish Peroxidase (Polyclonal), Goat | 1/10000 | Western Blot | Jackson ImmunoResearch Labs | 111-035-003 |
| Syndecan-1 (D4Y7H), Rabbit | 1:1000 | Western Blot | Cell Signaling Technology | 129122 |
| Transferrin (E7F47), Rabbit | 1:1000 | Western Blot | Cell Signaling Technology | 35293 |
